# Notch1 signaling pathway promotes growth and metastasis of gastric cancer via modulating CDH5

**DOI:** 10.18632/aging.206061

**Published:** 2024-08-21

**Authors:** Lingshan Zhou, Yuan Yang, Yuwei Ye, Qian Qiao, Yingying Mi, Hongfang Liu, Ya Zheng, Yuping Wang, Min Liu, Yongning Zhou

**Affiliations:** 1 Department of Gastroenterology, The First Hospital of Lanzhou University, Lanzhou 730000, China; 2 Department of Geriatrics Ward 2, The First Hospital of Lanzhou University, Lanzhou 730000, China; 3 Department of Gastroenterology, The First Affiliated Hospital, Hengyang Medical School, University of South China, Hengyang 421001, Hunan, China; 4 Department of Gastroenterology Ward 2, Shanxi Provincial People’s Hospital, Xian 710000, China; 5 The First Clinical Medical College, Lanzhou University, Lanzhou 730000, China

**Keywords:** Notch1, CDH5, gastric cancer, migration, invasion

## Abstract

Objective: To explore the underlying molecular mechanism of Notch1/cadherin 5 (CDH5) pathway in modulating in cell malignant behaviors of gastric cancer (GC).

Methods: We performed bioinformatic analyses to screen the potential target genes of Notch1 from cadherins in GC. Western blot and RT-PCR were conducted to detect CDH5 expression in GC tissues and cells. We utilized chromatin immunoprecipitation (CHIP) assays to assess the interaction of Notch1 with CDH5 gene. The effects of Notch1/CDH5 axis on the proliferation, invasion, migration and vasculogenic mimicry in GC cells were evaluated by EdU, wound healing, transwell, and tubule formation assays.

Results: Significantly increased CDH5 expression was found in GC tissues compared with paracancerous tissues and associated to clinical stage and poor overall survival (OS) in patients with GC. Notch1 positively regulate the expression of CDH5 in GC cells. CHIP assays validated that CDH5 was a direct target of Notch1. In addition, Notch1 upregulation enhanced the proliferation, migration, invasion and vasculogenic mimicry capacity of GC cells, which could be attenuated by CDH5 silencing.

Conclusions: These results indicated Notch1 upregulation enhanced GC malignant behaviors by triggering CDH5, suggesting that targeting Notch1/CDH5 axis could be a potential therapeutic strategy for GC progression.

## INTRODUCTION

Gastric cancer (GC) is one of the most common malignancies worldwide, accounting for approximately 1,089,103 new cases and 768,793 deaths in 2020 [1]. Eastern Asia has the highest incidence rate [1]. Despite great progress have made in the diagnosis and multimodal therapy, GC still ranks second for mortality of cancer in China and majority of patients with GC are detected at advanced stages [2]. Invasion and metastasis are important vitally important causes of mortality in GC patients. It has been reported that distant metastasis was responsible for over 90% mortality of GC patients [3]. Hence, it is imperative to search for novel therapeutic targets for GC invasion and metastasis.

The Notch pathway is involved in determination of cell fate, including cell differentiation, proliferation, migration and apoptosis [4]. Activation of the Notch signalling pathway requires interaction with ligands, resulting in the protein cleavage and release of Notch intracellular domain (NICD) [5]. NICD is translocated into the nucleus and interacts with various transcriptional activation complexes, like the DNA binding protein recombination signal binding protein-JK (RBPJ), leading to triggering the transcription of downstream target genes [6]. Accumulating evidence shows that Notch signalling pathway plays a crucial role in cancer. Notch1 upregulation in GC related to the poor prognosis has been broadly reported [7, 8]. Additionally, the significantly increased expression of Notch1 was correlated with lymph node metastasis of GC [8]. Similarly, a meta-analysis showed that the expression of Notch1 was higher in GC tissues compared to normal tissues, which was associated with lymphovascular invasion and distal metastasis [9]. Many studies have also verified that Notch1 signalling could promote the migration and invasion of GC cells [10, 11]. Thus, Notch1 offers a promising therapeutic target for managing GC. Unfortunately, targeting Notch1 simultaneously poses significant risks, such as gastrointestinal toxicity, diminishment of normal stem cell populations and increased risk of cancers in which Notch1 acts as a tumor suppressor. These risks become a huge obstacle to the clinical application of Notch1 inhibitors [12, 13]. Therefore, exploring the downstream target genes of Notch1 signalling exhibits important significance for GC therapy.

During occurrence and development of tumors, the signals related to cell adhesion are often abnormally modulated, resulting in the loss of cell contact inhibition and changes in cell migration and interstitial interaction. The cadherins are a major class of cell-cell adhesion molecules and have been widely studied in cancers [14]. The epithelial-mesenchymal transition (EMT) is thought to be the initiation of cancer cell invasion and metastasis. E-cadherin (CDH1) is a vital player in this process and considered to be a tumor suppressor [15]. Aberrant expression of N-cadherin occurred in many cancers and is closely related to aspects of malignant tumor progression, including invasion and metastasis [16]. Apart from E- and N-cadherin, the relevance of other cadherins has been reported in cancer progression and metastasis, such as cadherin 5 (CDH5, VE-cadherin), cadherin 6 and cadherin 17 [17]. Therefore, cadherins play a critical role in the regulation of cancer invasion and metastasis. Despite studies describing the association between cadherins and cancer progression, whether cadherins are involved in Notch1 signalling pathways and their pathological implications in GC progression and metastasis remain poorly investigated.

In this study, we used the databases to predict that CDH5 might be the target gene of Notch1. Subsequently, we demonstrated that CDH5 expression was significantly elevated in GC tissues compared to normal tissues, correlating with poor prognosis in patients with GC. Importantly, our study elucidated the positive regulatory role of Notch1 on CDH5 expression, establishing CDH5 as a direct target of Notch1. Moreover, Notch1 could promote the proliferation, migration, invasion and vasculogenic mimicry in GC cells, which could be attenuated by CDH5 silencing. Our results provided new insights into the mechanisms of Notch1 in invasion and metastasis of GC.

## RESULTS

### Bioinformatics screening of the target genes of Notch1 in CDH family members

**Figure 1:**
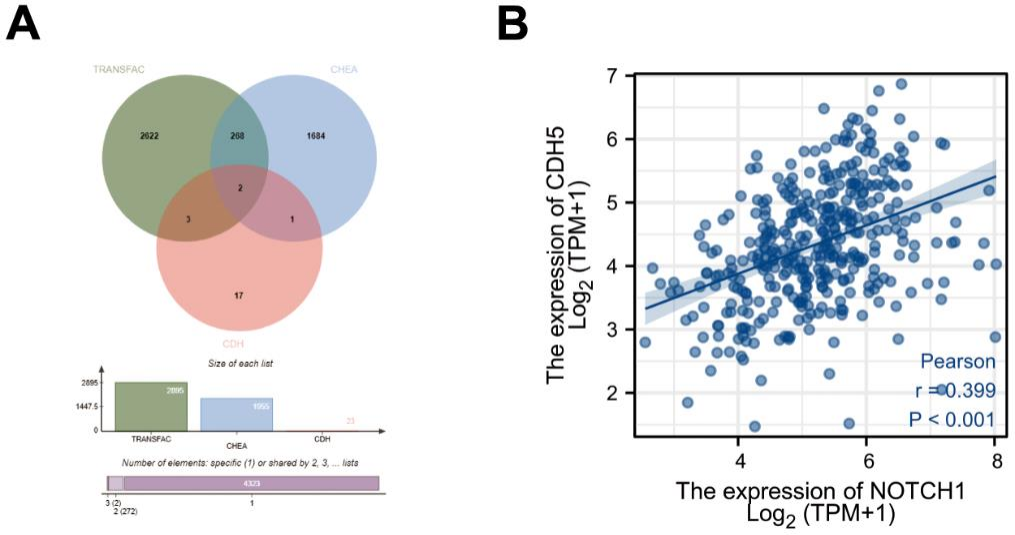
**Bioinformatics screening of target gene of Notch1 in gastric cancer.** (**A**) Workflow of target gene identification in the TRANSFAC database, ChEA database and CDH members. (**B**) Pearson’s correlation between expression of Notch1 and CDH5.

As mentioned above, Notch1 often works in combination with transcription factor RBPJ to activate the downstream target genes. Thus, we explored the potential target genes of RBPJ through the TRANSFAC database (http://transfac.gbf.de/) and CHIP Enrichment Analysis (ChEA) database (http://amp.pharm.mssm.edu/lib/chea.jsp) [18, 19]. A total of 23 CDH members were acquired from the literature [20]. Finally, we obtained 2 candidate genes (CDH5 and CDH24) after taking the above intersection (Figure 1A). According to the previous research [14, 16], we selected CDH5 for further study. The transcriptome profiling and corresponding clinical data regarding 375 GC and 32 adjacent normal samples were downloaded from the TCGA (https://portal.gdc.cancer.gov). The correlation of Notch1 and CDH5 gene expression was investigated using Pearson’s correlation analysis. The result showed a positive correlation between Notch1 and CDH5 (Figure 1B).

### Increased expression of CDH5 was observed in GC tissues and associated with poor overall survival (OS) in patients with GC

**Figure 2:**
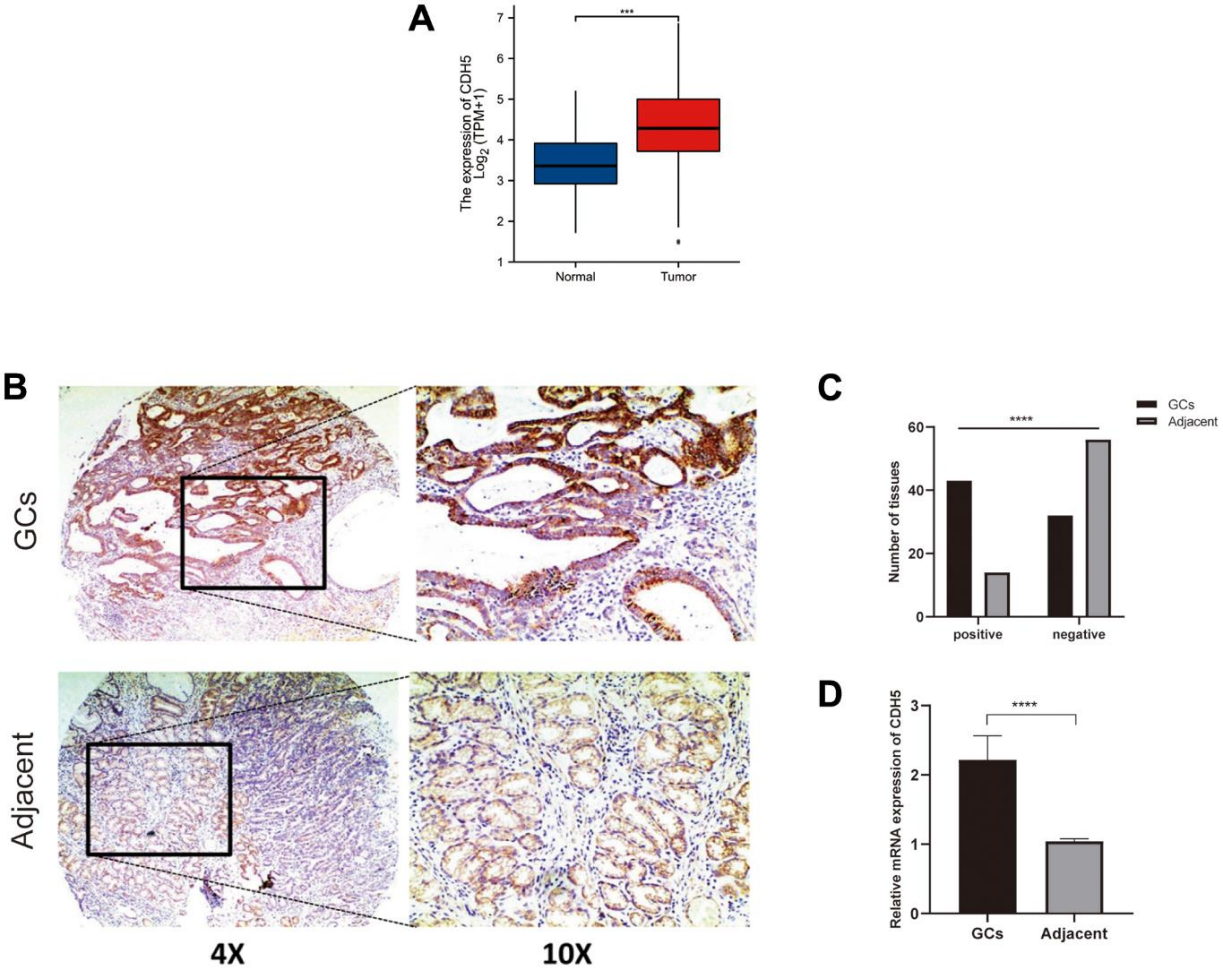
**CDH5 expression was upregulated in gastric cancer tissues.** (**A**) Expression level of the CDH5 gene in the GC samples compared to normal tissues through GEPIA. (**B**) Representative photomicrographs of IHC staining show CDH5 expression in gastric cancer tissues and adjacent normal breast tissues in tissue microarrays. Magnification×40 (left) and ×100 (right). (**C**) The difference in CDH5 expression levels between GC tissues and adjacent tissues in tissue microarrays. (**D**) The difference in mRNA expression levels of CDH5 between GC tissues and adjacent tissues form 32-pair clinical tissue samples. **** P<0.0001.

**Figure 3:**
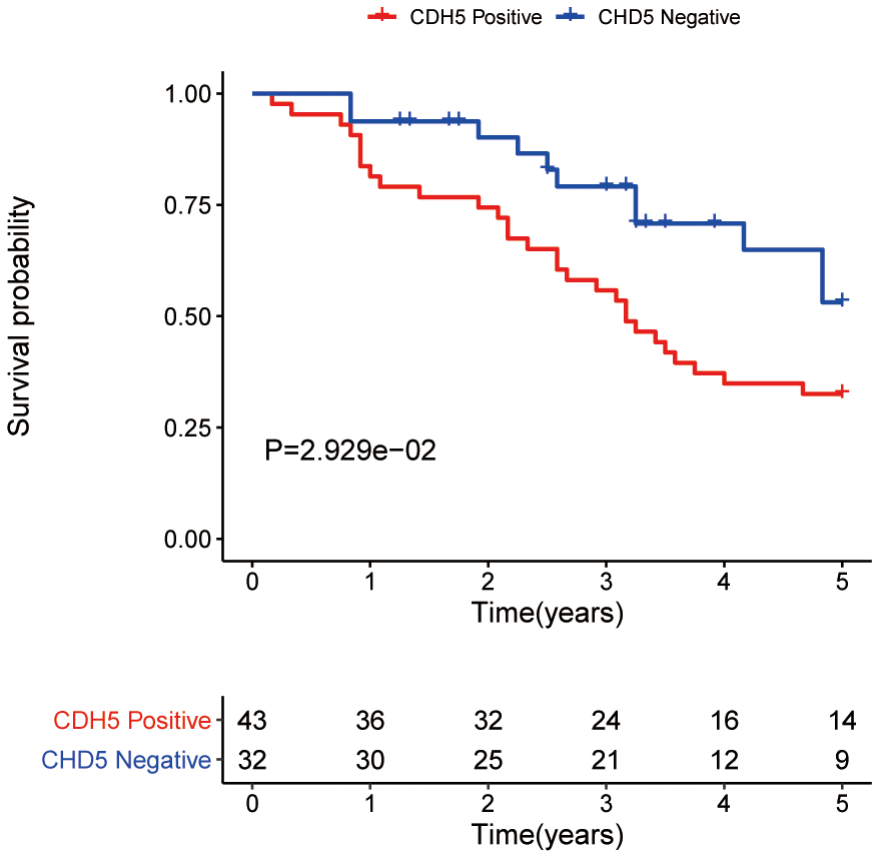
Overall survival analysis of gastric cancer patients with positive and negative CDH5 expression in the tissue microarrays.

Through GEPIA, we explored the differential expression of CDH5 between GC and adjacent normal samples and found the expression of CDH5 was higher in GC samples (Figure 2A). Then, we further used tissue microarray to validate the expression of CDH5 in GC. As expected, the proportion of CDH5-positive tissues was significantly increased in GC tissue samples compared with normal tissues (P <0.05) (Figure 2B, 2C). RT-PCR revealed CDH5 mRNA level was markedly increased in GC tissues in comparison with the control (Figure 2D). Additionally, we found that the overexpression of CDH5 in GC tissues was correlated with TNM stage (P<0.05) (Table 1). The Kaplan-Meier survival curves showed that CDH5-positive patients had a worse prognosis than CDH5-negative patients (Figure 3).

**Table 1:** Correlation between CDH5 expression with clinicopathological parameters of gastric cancer patients (n=75) from gastric cancer tissue microarrays.

**Parameters**	**CDH5 expression**			**P-value**
	**Positive**	**Negative**	**Total**	
gender				0.983
Female (%)	12	9	21	
Age (years)				0.193
<60	24	13	37	
≥60	19	19	38	
Stage				0.045
I-II	13	17	30	
III-IV	30	15	45	
T				0.193
1-2	9	11	20	
3-4	34	21	55	
N				0.294
0-1	15	15	30	
2-3	28	17	45	
M				0.855
0	37	28	65	
1	6	4	10	

### Notch1 positively regulated CDH5

**Figure 4:**
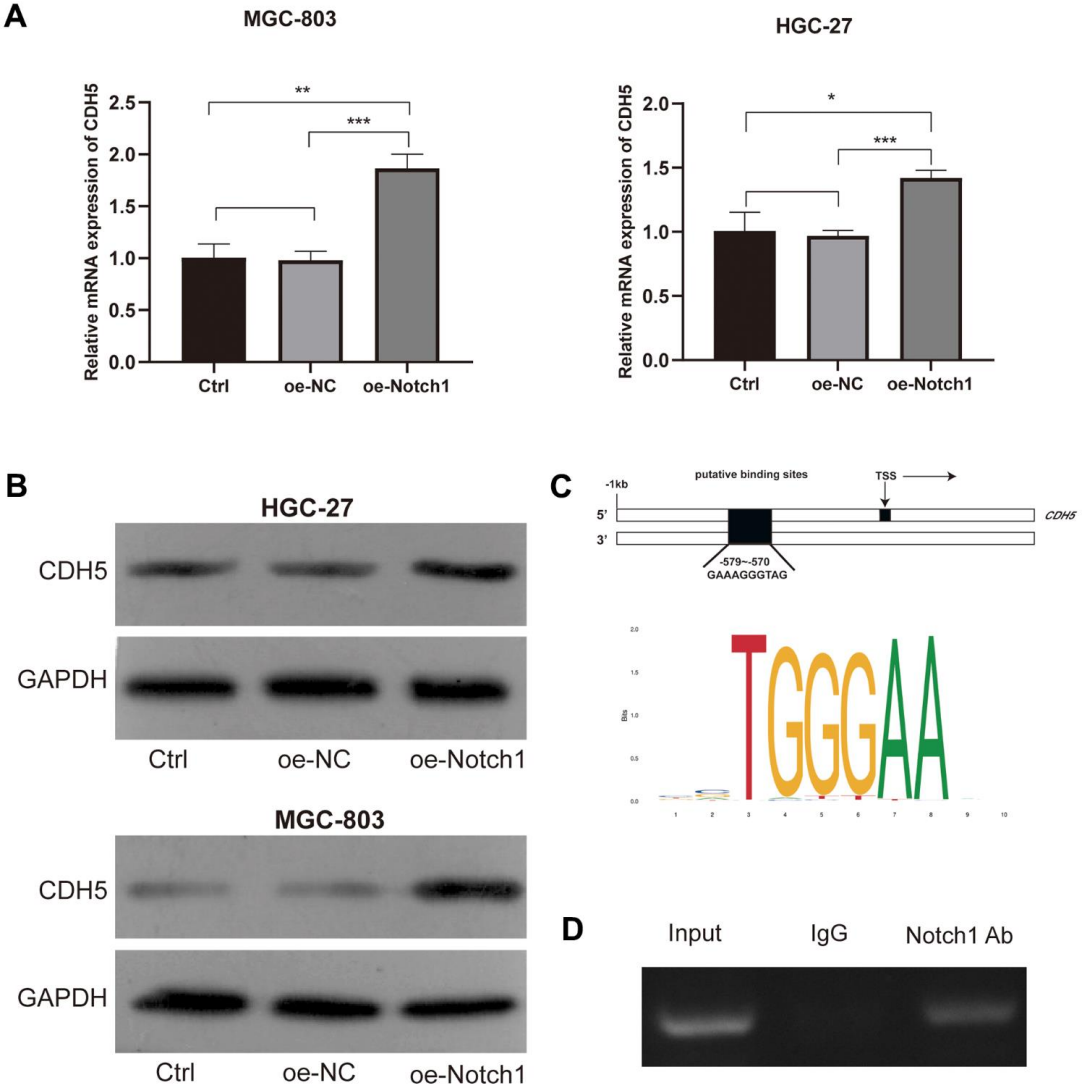
**Notch1 positively regulated the expression of CDH5.** (**A**) Notch1 positively regulated mRNA levels of CDH5 in MGC-803 (upper) and HGC-27 cells (lower). (**B**) Notch1 positively regulated protein levels of CDH5 in MGC-803 (upper) and HGC-27 cells (lower). (**C**) Schematic diagram of putative binding sites (−579 to −570) within the CDH5 promoter. (**D**) CHIP assay showed Notch1 bind to the CDH5 promoter. Input - input fraction, IgG - sample incubated with rabbit IgG, Notch1 Ab – sample incubated with anti-Notch1 antibody.

In order to investigate the relationship between Notch1 and CDH5, RT-PCR and WB assays were employed and confirmed that Notch1 could positively regulated CDH5 expression (Figure 4A, 4B). To study whether Notch1 could bind to CDH5 promoter, further inducing CDH5 transcription, we scanned the 1 kb sequences upstream from the CDH5 transcription start site (TSS). According to the bioinformatic analysis, there is a potential binding site at −579 to −570 bp. The schematic representation is shown in Figure 4C. Next, we performed the CHIP assay and detected more significant enrichment of the Notch1-bound specific CDH5 promoter fragments related to the IgG antibody group, indicating that CDH5 could be a direct target of Notch1 (Figure 4D).

### Activated Notch1 induced CDH5 to facilitate GC cell proliferation, migration, invasion and vasculogenic mimicry

**Figure 5:**
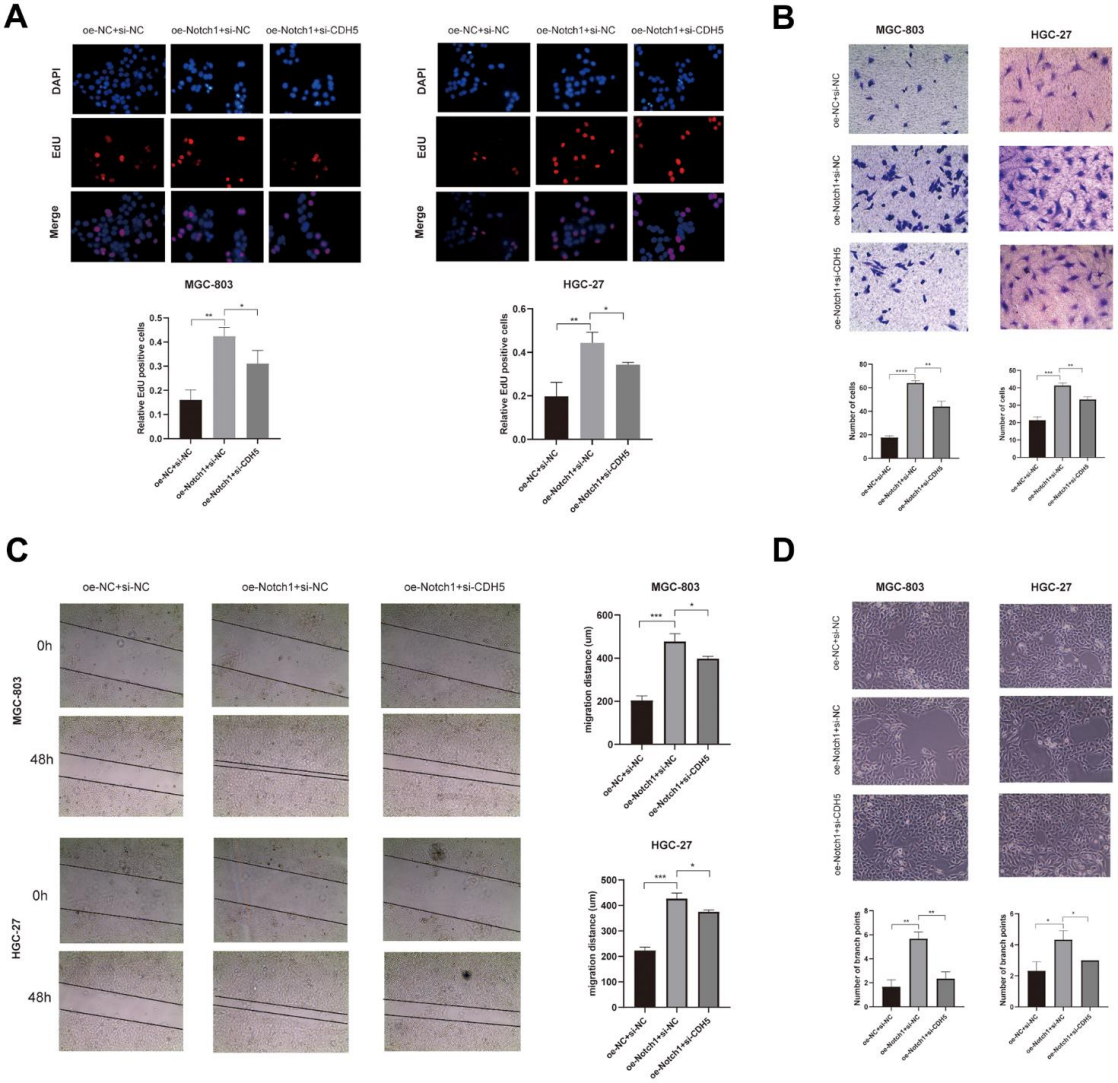
**Overexpressed Notch1 promoted gastric cancer cell growth, invasion and migration with CDH5 involvement.** (**A**) EdU assay in MGC-803 and HGC-27 cells transfected with NC, Notch1-lentivirus, and Notch1-lentivirus+si-CDH5. (**B**) Transwell assay in MGC-803 and HGC-27 cells transfected with NC, Notch1-lentivirus, and Notch1-lentivirus+si-CDH5. (**C**) Scratch wound healing assays in MGC-803 and HGC-27 cells transfected with NC, Notch1-lentivirus, and Notch1-lentivirus+si-CDH5. (**D**) Tubule formation assays in MGC-803 and HGC-27 cells transfected with NC, Notch1-lentivirus, and Notch1-lentivirus+si-CDH5.

To get more insight into the regulatory role of Notch1 in GC cell migration and invasion, we investigated whether Notch1 is also responsible for GC cell behaviors in the silencing of CDH5. All cells were classified into 3 groups: si-NC+oe-NC, oe-Notch1+ si-NC, and oe-Notch1+si-CDH5. The results showed that the Notch1 upregulation significantly promoted the proliferation, migration, invasion, and vasculogenic mimicry capacity of gastric cancer cells (Figure 5A–5D). And the silencing of CDH5 could attenuate these effects conferred by Notch1 upregulation (Figure 5A–5D). Thus, our results suggest that the upregulation of Notch1 enhanced GC cell malignant biological properties by positively regulating CDH5.

## DISCUSSION

The process of invasion and metastasis of GC cells is not fully understood and effective therapies are still lacking. Evidence has revealed the core role of Notch1 signalling in tumor malignant behavior [21, 22]. To better understand the underlying mechanism of Notch1 signaling in GC cell, we performed the current study and found that Notch1 enhanced GC cell malignant biological properties by positively regulating CDH5. To our knowledge, this is the first study to explore the mechanism regarding controlling GC progression by linking Notch1 pathway and its downstream gene CDH5.

Aberrant activation of Nocth1 signalling pathway has been reported in GC, which plays an important role in tumor progression [9, 23]. Previous studies showed that Notch1 can promote invasion and metastasis in many tumor cells, including GC cells [10, 11, 24]. This is in line with our findings in gastric cancer cell lines MGC803 and HGC27. At the same time, Zhang, et al. found that downregulation of Notch1 inhibited invasion and metastasis of GC cells [25]. Considering available evidence, Notch1 offers a potential therapeutic target in future strategies for combating GC. However, Notch1 signalling pathway is a complex pathway with multiple biological functions, with many existing obstacles for its clinical application [13]. So, we explored the downstream target gene of Notch1.

By performing bioinformatic analysis, we identified CDH5 and CDH24 from CDH family as candidate target genes of Notch1. It has been reported that protein expression of CDH5 was elevated in GC tissues [26]. For this reason, CDH5 was selected for further analysis in this study. Likewise, in Chinese population, we found that the CDH5 expression level was higher in GC tissues compared to normal tissues. And the CDH5 upregulation in GC was related to the worse clinical stage and poor prognosis. Current study indicated that CDH5 played a vital role in vasculogenic mimicry (VM) [27]. VM, a unique blood supply pattern independent of endothelial vessels in tumor cells, is closely associated with invasion, metastasis and poor prognosis [27]. Aberrant expression of CDH5 has been observed in cancer and was associated with VM [28]. The upregulated expression of CDH5 promoted VM formation, resulting in enhancing the migration and invasion capacity of GC cells [29]. These findings suggested that CDH5 played an important role in GC progression and may be a potential target for GC.

Notch is responsible for the development of vascular networks in embryonic stages. There exists a positive relationship between Notch with VM in cancer [30]. In melanoma, CDH5 expression was associated with the activation of the Notch pathway [27]. In GC cells, we also found that the activation of Notch1 could promote the transcription of CDH5. A previous study revealed that Notch1 could affect tumor metastasis by regulating CDH family members, such as E-cadherin [31]. It has been reported that the transcription factor can enhance activity of CDH5 by binding CDH5 promoter [32]. In this study, we also demonstrated that Notch1 could bind to CDH5 promoter through the CHIP assay. Furthermore, we found that the effect of Notch1 on the invasion and migration of GC cells partly depended on CDH5 by rescue assay. Therefore, Notch1 could bind to CDH5 promoter to upregulate the expression of CDH5, thus enhancing the migration and invasion capacity of GC cells.

In this study, we showed that Notch1 could regulate CDH5 expression by targeting CDH5 promotor, providing insight into how Notch1 upregulation enhanced the proliferation, migration, invasion, and vasculogenic mimicry capacity of gastric cancer cells. These findings advanced our understanding of the molecular mechanisms underlying GC progression and suggested potential therapeutic strategy for GC by targeting Notch1/CDH5 axis.

## MATERIALS AND METHODS

### Tissue microarrays

A human GC tissue microarray containing 75 tumor tissues and 70 adjacent normal tissues from patients with GC were purchased from Outdo Biotech Company, (Shanghai, China), which also provided detailed clinicopathological information of these GC patients.

### Immunohistochemistry

The tissue sections underwent immunohistochemical staining using a primary antibody to CDH5 (CST, USA) at a dilution of 1: 500. The scores were obtained by the percentage of stained cells and intensity of immune staining. The scores of percentages of CDH5-positive cells were assigned as follows: 0, <10% positive cells; 1, 11–25% positive cells; 2, 26–50%, positive cells; 3, >50% positive cells. A total score >3 was considered to be positive.

### Collection of GC samples

Our research group collected 32 pairs of GC tissues and their paracancerous tissues that came from the Department of Surgical Oncology in the First Hospital of Lanzhou University. All cases were diagnosed by pathology as gastric cancer. The included patients did not undergo preoperative radiotherapy or chemotherapy and had no other types of cancer.

### Cell line and culture

Human GC cell lines (MGC803 and HGC27) were purchased from the American Type Culture Collection (USA). Both cells were cultured in RPMI-1640 medium (Meilun, Dalian, China), supplemented with 10% fetal bovine serum (Minhai, Lanzhou, China) and 1% antibiotics (streptomycin/penicillin). Both of them were maintained in a 37° C incubator containing 5% CO_2_.

### Lentivirus stable transfection

To generate Notch1 intracellular domain (Notch1-IC)-labeled GC cells, we customized the lentiviral vector, pLV-CMV-MCS-3FLAG-Luciferase-T2A-Notch1 expressing luciferase and Notch1-IC from Hanbio (Shanghai, China). The cells stably overexpressing Notch1-IC were selected using puromycin. The efficiency of Notch1-IC overexpression was confirmed by Western blot analysis.

### RT-PCR

Total RNA was isolated from cells or samples with TRIzol (Invitrogen, USA) and then used for the synthesis of the cDNA with the reverse transcription assay kit (Invitrogen, USA), following the manufacturer’s protocol. Then, the cDNAs were amplified with primers.

Primers targeting Notch1 (NICD) (Forward 5′-CGC ACA AGG TGT CTT CCA GAT CC-3′, Reverse 5′-CGT CGG CGT GTG AGT TGA TGA G-3′) and CDH5 (Forward 5′-CCT CTG TGG GCT CTC TGT TTG TTG -3′, Reverse 5′-CTC AAT GGT GAA AGC GTC CTG GTA G-3′) were utilized in the experiments.

### Western blot

RIPA buffer supplemented with protease inhibitor (Thermo Fisher Scientific, USA) was used to extract protein. Protein concentration was determined using the BCA protein assay kit (Solarbio, Beijing, China). After being boiled at 95° C for 5 min, protein samples were isolated by 10% SDS-PAGE and then transferred onto PVDF (Millipore, USA) membranes. After overnight incubation with primary antibodies, they were washed three times and then incubated with a secondary antibody for 1 h. Membranes were developed with ECL substrate (Beyotime Biotechnology, Shanghai, China) and exposed using film.

### Cell proliferation assay

The cell proliferation was assessed by using Meilun EdU Cell Proliferation Kit with Alexa Fluor555 (Meilun, Dalian, China), according to the manufacturer’s instructions. GC cells with different transfections were seeded in 96-well plates and incubated with 50 μM EdU for 2 h at 37° C. Cells were fixed with 4% paraformaldehyde and stained cell nuclei with 5 μg/mL Hoechst 33342. The EdU cell line was observed and photographed using the Olympus IX51 microscope (Olympus, Tokyo, Japan).

### Scratch wound healing assays

GC cells with different transfections were seeded in 6-well plates. Cells monolayers were scraped with the fine end of 10-μL pipette tips. Images of migrated cells were observed under phase-contrast microscopy. The distances between the two edges were measured at 0 and 48 hours.

### Invasion assays

The transwell chamber (Costar, USA) was precoated with Matrigel matrix. Cells were placed in the upper chamber with serum-free DMEM. And 10% FBS-supplemented DMEM was added in the lower chamber. After incubation for 24h, cells that invaded the lower surface were fixed and stained. The numbers of invaded cells were counted using bright-field microscopy.

### Tube formation assays

50 μL of Matrigel (BD Biosciences, USA) was added to each well of 96-well plate and incubated for 30 min. A total of 2×10^4^ cells were seeded onto the top of the Matrigel matrix. After incubating overnight at 37° C, pictures were captured under a microscope.

### CHIP assay

GC cells were cross-linked with a 1% formaldehyde solution. Then, the samples were lysed to obtain DNA fragments <200 by ultrasonic disruption. IP was performed using anti-Notch1-IC or rabbit IgG (CST, USA) antibody. The obtained DNA fragments were incubated with the antibodies overnight at 4° C. The immunoprecipitated DNA was used for subsequent PCR with primer encompassing the putative binding sites (Forward 5′-GCC CCA GAG CTT GAT TTT CT -3′, Reverse 5′-AGA GCT TCT GTC CCT TTC CC -3′).

### Statistical analysis

Statistical analyses were performed using SPSS 24. The differences between the two groups were assessed by the student’s t-test. Categorical data were analyzed using the Chi-square test. *P*-value less than 0.05 was considered statistically significant.
